# Modifications of myofilament protein phosphorylation and function in response to cardiac arrest induced in a swine model

**DOI:** 10.3389/fphys.2015.00199

**Published:** 2015-07-16

**Authors:** Mike Woodward, Michael J. Previs, Timothy J. Mader, Edward P. Debold

**Affiliations:** ^1^Molecular and Cellular Biology Graduate Program, University of MassachusettsAmherst, MA, USA; ^2^Department of Molecular Physiology and Biophysics, University of VermontBurlington, VT, USA; ^3^Department of Emergency Medicine, Baystate Medical Center/Tufts University School of MedicineSpringfield, MA, USA; ^4^Muscle Biophysics Lab, Department of Kinesiology, University of MassachusettsAmherst, MA, USA

**Keywords:** cardiac arrest, resuscitation, myosin, troponin, phosphorylation, motility

## Abstract

Cardiac arrest is a prevalent condition with a poor prognosis, attributable in part to persistent myocardial dysfunction following resuscitation. The molecular basis of this dysfunction remains unclear. We induced cardiac arrest in a porcine model of acute sudden death and assessed the impact of ischemia and reperfusion on the molecular function of isolated cardiac contractile proteins. Cardiac arrest was electrically induced, left untreated for 12 min, and followed by a resuscitation protocol. With successful resuscitations, the heart was reperfused for 2 h (IR2) and the muscle harvested. In failed resuscitations, tissue samples were taken following the failed efforts (IDNR). Actin filament velocity, using myosin isolated from IR2 or IDNR cardiac tissue, was nearly identical to myosin from the control tissue in a motility assay. However, both maximal velocity (25% faster than control) and calcium sensitivity (pCa_50_ 6.57 ± 0.04 IDNR vs. 6.34 ± 0.07 control) were significantly (*p* < 0.05) enhanced using native thin filaments (actin+troponin+tropomyosin) from IDNR samples, suggesting that the enhanced velocity is mediated through an alteration in muscle regulatory proteins (troponin+tropomyosin). Mass spectrometry analysis showed that only samples from the IR2 had an increase in total phosphorylation levels of troponin (Tn) and tropomyosin (Tm), but both IR2 and IDNR samples demonstrated a significant shift from mono-phosphorylated to bis-phosphorylated forms of the inhibitory subunit of Tn (TnI) compared to control. This suggests that the shift to bis-phosphorylation of TnI is associated with the enhanced function in IDNR, but this effect may be attenuated when phosphorylation of Tm is increased in tandem, as observed for IR2. There are likely many other molecular changes induced following cardiac arrest, but to our knowledge, these data provide the first evidence that this form cardiac arrest can alter the *in vitro* function of the cardiac contractile proteins.

## Introduction

Sudden cardiac arrest is typically precipitated by ventricular fibrillation or pulseless ventricular tachycardia, a life-threatening condition characterized by an abrupt loss of blood flow resulting in progressive global ischemia (Neumar et al., 2008). Successful resuscitation with return of spontaneous circulation can restore blood flow to near normal levels within minutes of an event, however the prognosis for revived patients remains quite poor with only ~10% surviving to hospital discharge if it occurs outside of a hospital (Schoenenberger et al., 1994; Eisenberg and Mengert, 2001) and this only improves to 23% if it occurs in a hospital setting (Roger et al., 2012). After resuscitation many patients experience “postcardiac arrest syndrome,” characterized by a persistent mechanical myocardial dysfunction in the absence of large morphological changes (Kern, 2002), which is believed to be a leading cause of the high morbidity and mortality following cardiac arrest (Neumar et al., 2008). However, the molecular basis of the persistent mechanical dysfunction is unclear and thus the means to improve the extremely poor prognosis is unclear (Bolli and Marban, 1999; Chalkias and Xanthos, 2012). For example it is unknown whether cardiac arrest directly affects the function of contractile proteins that give rise to the force and power generating capacity of the heart.

At a molecular level, force and power in the heart result from the calcium dependent sliding of actin-based thin filaments past myosin-thick filaments in a process driven by the hydrolysis of adenosine triphosphate (ATP). The earliest biochemical changes that occur in the myocytes during an ischemic event are the accumulation of metabolites including: hydrogen ions (H^+^), inorganic phosphate (P_i_), and adenosine diphosphate (ADP) (Allen and Orchard, 1987; Schaefer et al., 1990; Elliott et al., 1992; Bolli and Marban, 1999). These molecules exert direct depressive effects on the mechanical function of the contractile apparatus, however levels of these molecules quickly (<5 min) returned to normal with return of blood flow and therefore cannot explain the persistent decrease in cardiac function (Allen and Orchard, 1987; Schaefer et al., 1990; Elliott et al., 1992; Bolli and Marban, 1999). Thus, the more likely cause of post-arrest myocardial dysfunction are the persistent structural modifications of the contractile apparatus similar to those which occur in models of localized ischemia (Rao et al., 2007). However, to our knowledge neither structural modifications nor functional consequences have been identified in global ischemic event such as cardiac arrest (Chalkias and Xanthos, 2012).

Models of more localized and cardiac ischemia have shown that myosin, actin and thin filament calcium-dependent regulatory proteins, troponin (Tn) and tropomyosin (Tm), which regulate actomyosin interactions can undergo post-translational modifications including truncation (Westfall and Solaro, 1992; Gao et al., 1997; van Eyk et al., 1998; McDonough et al., 1999; van Eyk and Murphy, 2001; Foster et al., 2003; Day et al., 2007) and phosphorylation (Rao et al., 2007, 2009; Han and Ogut, 2010, 2011) which impact myofilament function. Due to the known consequences of localized ischemia on contractile function, we explored the time-dependent effects of global ischemia and reperfusion on myofilament structure and function in a porcine model. Here, we determined the impact of a 12-min bout of cardiac ischemia via arrest and attempted myocardial resuscitation in a porcine model on the structural and functional characteristics of cardiac myosin and native thin filaments. We found that the global ischemia and subsequent reperfusion had no effect on the ability of myosin to move actin or native thin filaments in an *in vitro* motility assay, regardless of the outcome of resuscitation. While native thin filament calcium sensitivity and velocity were not perturbed when resuscitation was successful, calcium sensitivity and velocity of native thin filaments was significantly enhanced in tissue from failed resuscitation. Protein analysis by quantitative mass spectrometry demonstrated increases in phosphorylation of both the inhibitory subunit of Tn (TnI) and α-Tm associated with global ischemia and reperfusion, in the absence of truncation of TnI. In conjunction with the functional analyses, these results suggest that the increase in native thin filament calcium sensitivity and sliding velocity could be attributed to an increase in the phosphorylation of TnI in the absence of accompanying changes in α-Tm. These data provide the first assessment of the impact of global ischemia and resuscitation on the structure and function of the heart's molecular motor myosin and its thin filament binding partner.

## Methods

### Ischemia/reperfusion protocol

All surgical procedures involving animals were in strict compliance with the NIH Guide for the Care and Use of Animals. The parent study from which the tissue samples were obtained was IACUC-approved and conducted in a USDA certified laboratory. Female domestic Yorkshire swine aged 3–4 months and weighing approximately 30–35 kg prepared as previously described (Mader et al., 2010), were used for the cardiac arrest-resuscitation experiments. The three animals included in this substudy were part of a larger study involving 80 animals, 45% of which attained ROSC. During the protocol the animals were sedated with intramuscular telazol (5 mg/kg), ketamine (2.5 mg/kg), and xylazine (2.5 mg/kg). Isoflurane was provided to facilitate endotracheal intubation and intravenous (IV) access. The inhalation anesthetic was then discontinued and a surgical plane of anesthesia was achieved using an IV propofol bolus (2 mg/kg) followed by a continuous infusion (80 mics/kg/min) titrated to effect. The animals were ventilated with room air, using a volume-cycled ventilator adjusted the tidal volume and ventilatory rate to maintain eucapnea. A nasopharyngeal probe was placed through the oral cavity into the animal's esophagus to measure core body temperature. Three surface electrodes configured to correspond to a standard lead II electrocardiogram (ECG) surface electrodes were secured to the proximal forelimbs and thorax.

Neuromuscular paralysis was induced with pancuronium (4 mg initial bolus IV) and an arterial introducer (8.5 Fr) was placed into the right femoral artery and a venous introducer (8.5 Fr) into the right femoral vein under direct visualization. Micro–manometer tipped pressure catheters (Mikro-Tip, Millar Instruments, Houston, TX) were placed into the ascending aorta and right atrium. Arterial blood gas was obtained as soon as access was established and just prior to ventricular fibrillation (VF) induction. All central vascular access ports were connected to a pressurized liter bag of normal saline containing heparin. The ECG tracing, as well as the arterial and venous pressures were monitored and recorded continuously throughout the experiment (PowerLab M8/30, AD Instruments, Colorado Springs, CO). Immediately before induction of VF, a 2-mg bolus of pancuronium was given, the propofol infusion was discontinued, and the ventilator was disconnected.

Global ischemia and the resuscitation protocol were performed as previously described (Mader et al., 2010). Briefly, VF was induced by a 3-s, 60-Hz, 100-mA transthoracic alternating current. At minute 12 of untreated VF, dynamic baseline characteristics were again recorded and resuscitation was attempted beginning with mechanical chest compressions using an oxygen-powered mechanical resuscitation device (Life-Stat Mechanical CPR System, Michigan Instruments, Grand Rapids, MI) that provides standardized closed chest compressions in the anterior-posterior direction at a rate of 100/min. The device was programmed to deliver chest compressions and ventilation (*V_t_* = 500 cc, F_i_0_2_ = 100%) in a ratio of 30:2.

**Figure 1:**
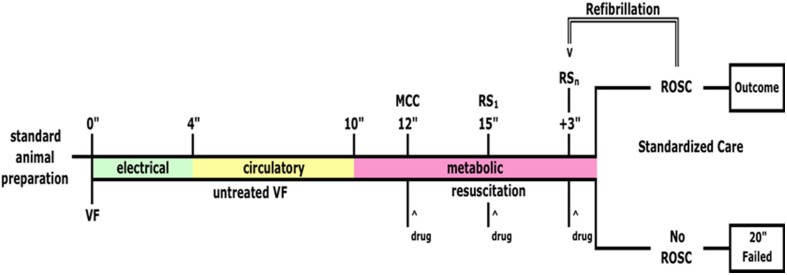
**Global ischemia protocol**. Ventricular fibrillation (VF) was induced as previously described (Mader et al., 2010) with a transthoracic current (100 mA at 60 Hz) and was untreated for 12 min. VF lasted for 4 min before complete electrical failure was reached (green bar) at which point circulation stopped completely (yellow bar) and finally metabolic failure is reached at ~10 min. (pink bar). Resuscitation efforts began 12 min after induction of VF, initially with manual chest compressions (MCC) and infusion of epinephrine (drug, 0.1 mg/kg). At minute 15 we attempted to restart circulation electrically (RS_1_) and at 18 min. If spontaneous circulation returned (ROSC) it was maintained for 2 h and then the animals were sacrificed and cardiac muscle samples taken from the left ventricle (Ischemia + 2 h reperfusion). If ROSC was not restored then after 20 min cardiac muscle samples were taken from the LV (Ischemia + failed resuscitation). Control animals were prepared identically but did not receive the ischemia protocol (Control). ^ Indicates when a drug was administered.

The animals were resuscitated using a standard combination of resuscitation drugs (see Figure 1). Attempted resuscitation terminated after the return of spontaneous circulation (ROSC) or 20 min of failure. Animals attaining ROSC were immediately placed back on the ventilator, a low dose propofol infusion was restarted and titrated to optimize the effect, and norepinephrine was given intravenously to maintain a systolic blood pressure above 80 mm Hg for 2 h. At the conclusion of each experiment, the hearts were immediately excised, diced, and put into liquid nitrogen. The samples were kept on dry ice during transport and stored in a −80°C freezer.

Cardiac tissue samples were obtained from three pig hearts under three conditions: (1) samples from a control animal that was subjected to the surgery but that did not experience cardiac arrest or subsequent resuscitation and reperfusion (control); (2) from an animal subjected to 12 min of global ischemia and attempted resuscitation for 20 min that included epinephrine (0.01 mg/kg), vasopressin (0.5 U/kg), amiodarone (4 mg/kg), sodium bicarbonate (1.0 mEq/kg), and metoprolol (0.2 mg/kg) but spontaneous circulation failed to return (IDNR); and (3) from an animal that underwent 12 min of global ischemia and successful resuscitation after epinephrine (0.01 mg/kg), vasopressin (0.5 U/kg), amiodarone (4 mg/kg) on the third defibrillation attempt, followed by 2 h of sustained reperfusion (IR2).

### Proteins

Cardiac myosin was isolated from porcine ventricular tissue based on adaptation of a method previously established for mouse cardiac myosin (Tyska et al., 2000). Briefly a 2-mL aliquot of extraction buffer (300 mM KCl, 150 mM PO_4_, 20 mM EDTA, 5 mM MgCl_2_, pH 6.7, 3.3 mM ATP, and 5 mM DTT) was added to ~400 mg of porcine cardiac tissue and continuously homogenized for 12 min in a 2 mL dounce homogenizer. The solution was then centrifuged (5 min; 10,000 g; 4°C) and the supernatant was centrifuged once more for 20 min at 400,000 g. The myosin containing supernatant was then precipitated by 10-fold dilution with ddH_2_O for 60 min and then centrifuged for 10 min at 10,000 g, and 4°C. The supernatant was disregarded and the myosin pellet was gently rinsed with cold ddH_2_O. The pellet was homogenized in a minimum volume of a high salt buffer (25 mM imidazole, 600 mM KCl, 1 mM EGTA, 4 mM MgCl_2_, pH 7.4) and the concentration determined with a spectrophotometer (extinction coefficient of 0.55, Margossian and Lowey, 1982).

An additional purification was performed on a subset of the cardiac myosin using hydrophobic interaction chromatography (HIC) as previously described (Malmqvist et al., 2004) with minor modifications. Briefly, the isolated myosin was first dialyzed overnight at 4°C against 1.45 M ammonium sulfate (AmSO_4_) and then centrifuged (15 min; 10,000 g) to remove any aggregating proteins. The supernatant was loaded on to the HIC column (5 mL Toyo pearl ether–650 M, Tosoh Biosciences Inc., Grove City, OH) in 1.45 M AmSO_4_. The AmSO_4_ was reduced to 1.2 M causing the elution of the purified myosin, following a collection of proteins that weakly interacted with the column.

Native thin filaments (actin, Tn complex, and Tm) were isolated using an established protocol (Lehman et al., 1995) in which ~200 mg of tissue was exposed to thin filament extraction buffer (25 mM imidazole, 1 mM EGTA, 100 mM KCl, 4 mM MgCl_2_, 5 mM ATP, 10 mM DTT, pH 7.0) and homogenized for 12 min on ice (0°C). The homogenate was initially centrifuged (10,000 g, 4°C) for 5 min followed by a second centrifugation (40,000 g, 4°C) of the supernatant for 20 min to remove myosin and any bulk tissue. Subsequently, the supernatant was kept and centrifuged (200,000 g, 4°C) again for 45 min to pellet the thin filaments which were re-suspended in 300 μL of thin filament extraction buffer before a final clarification spin (40,000 g, 4°C) for 5 min to remove any remaining myosin. The supernatant was kept and then a final centrifugation was performed for 45 min at 200,000 g to collect the thin filaments. The resultant pellets were brought up in an extraction buffer (7.0 pH, 25 mM imidazole, 1 mM EGTA, 100 mM KCl, 4 mM MgCl_2_, 10 mM DTT) labeled tetramethylrhodamine isothiocyanate-phallodin (TRITC-phallodin) (Sigma-Aldrich Inc., St. Louis, MI) at a 1 μM. Chicken skeletal actin was also used to assess the cardiac myosin function and it was isolated from pectoralis muscle and fluorescently labeled with TRITC-phallodin.

### *In vitro* motility

The impact of the ischemia-reperfusion of contractile protein function was assessed using the *in vitro* motility assay as described (Debold et al., 2011, 2012). Briefly, isolated myosin (in a high salt buffer: 300 mM KCl, 25 mM imidazole, 1 mM EGTA, 4 mM MgCl_2_, pH 7.4, 10 mM DTT) was adhered to a nitrocellulose coated microscope coverslip as part of a flow cell at 100 μg/ml. Bovine serum albumin (0.5 mg/mL) was then added to cover any areas of the surface not coated by myosin. The native thin filaments were then added and allowed to incubate on the surface for 1 min in the absence of ATP. Finally a motility buffer was added to the chamber (25 mM KCl, 25 mM imidazole, 1 mM EGTA, 4 mM MgCl_2_, pH 7.4, 2 mM ATP, 10 mM DTT, with an amount of CaCl_2_ required to achieve the appropriate free [Ca^2+^]) and an oxygen scavenging system (glucose oxidase, catalase, and catalase) to slow photo-bleaching of the fluorescently labeled actin filaments.

The flow cells were then loaded onto a Nikon Eclipse Ti inverted microscope with a 100 X, 1.4 NA CFI Plan Apo objective and the fluorescent actin filaments were visualized by an ICCD camera (Stanford Photonics, Inc., Palo Alto, CA, USA). The video was captured by an Epix-LVDS frame grabber (Epix, Inc., Buffalo Grove, IL, USA) coupled to the ICCD camera. Piper Control™ 2.5 software (Stanford Photonics, Inc., Palo Alto, CA, USA) was used to capture three to four videos from each flow-cell at 10 frames s^−1^ for 30 s. The filament motions were digitized and tracked using an automated program (Celltrak®, Motion Analysis Corporation, Santa Rosa, CA). Four to five motility experiments using separate myosin and thin filament isolations were performed for each condition. Differences in actin and native thin filament sliding velocities among each condition were determined using a One-Way ANOVA followed by a Tukey's HSD *post-hoc* test. To determine the calcium sensitivity of the native thin filaments their velocities as a function of the –log of the calcium concentration (pCa) were fitted to the Hill equation:

$$V=V_{\text{max}}/(1+10^{n(\text{pCa}50-\text{pCa})})$$

using SigmPlot® 11.2 (Systat Software San Jose, CA). The hill equation derived the calcium concentration required to elicit half maximal filament velocity (pCa_50_) and the Hill coefficient (*n*) to gain insight into the cooperative behavior of activation.

### Mass spectrometry

In preparation for mass spectrometry denaturing SDS-PAGE was conducted with precast 12% bis-Tris polyacrylimide gels (BioRad, Life Science Research, Hercules, CA). The identified bands were excised from the gel, cut into small cubes, and placed into Eppendorf tubes. Residual stain and excess water was removed with a 50% acetonitrile solution and the gel slices were dried in a speed vacuum device as previously described (Previs et al., 2012). Next, one set of samples was rehydrated with 8 μL of alkaline phosphatase (Sigma–Aldrich Inc., St. Louis, MI) in 92 μL of ammonium bicarbonate and incubated for 18 h at 30°C, allowing the phosphatase to thoroughly impregnate the gel and dephosphorylate the protein samples. The samples were dried via a speed vacuum device and subsequently rehydrated with 2 μg of trypsin (Promega, Madison, WI) in 100 μL of ammonium bicarbonate and incubated for 18 h at 37°C. After 18 h of incubation 7 μL of 90% formic acid was added to deactivate the trypsin. The resultant peptides were extracted with 25 mM ammonium bicarbonate/50% acetonitrile solution, dried in a speed vacuum device, and reconstituted in 0.05% heptafluorobutyric acid.

Electrospray ionization liquid chromatography tandem mass spectrometry (LC-MS) was carried out in data dependent mass spectrometry mode using an LTQ ion trap mass spectrometer (Thermo Electron Corporation) coupled to a 1 mm C18 column as previously described (Weith et al., 2012). Initial SEQUEST searches were performed to identify peptides using the IPI human protein sequence database (v3.75) downloaded from the EMBL-EBI website. Subsequent searches only contained the pig cardiac TnI **(**A5X497) and pig α-Tm **(**P42639**)** sequences downloaded from UniProtKB. The degrees of site-specific TnI and α-Tm phosphorylation and truncation of the C-terminus of TnI were determined from the extracted ion currents for specific peptides of interest in the LC chromatograms using label free-proteomic strategies as described in the Results Section. Phosphorylation levels were determined using each of the five reference peptides listed for TnI and α-Tm in the Results Section in three samples from each group, before and after treatment with alkaline phosphatase. Statistical significance was determined from the individual measurements from each reference peptide using a Student's *t*-test.

## Results

### Changes to contractile protein function

**Figure 2:**
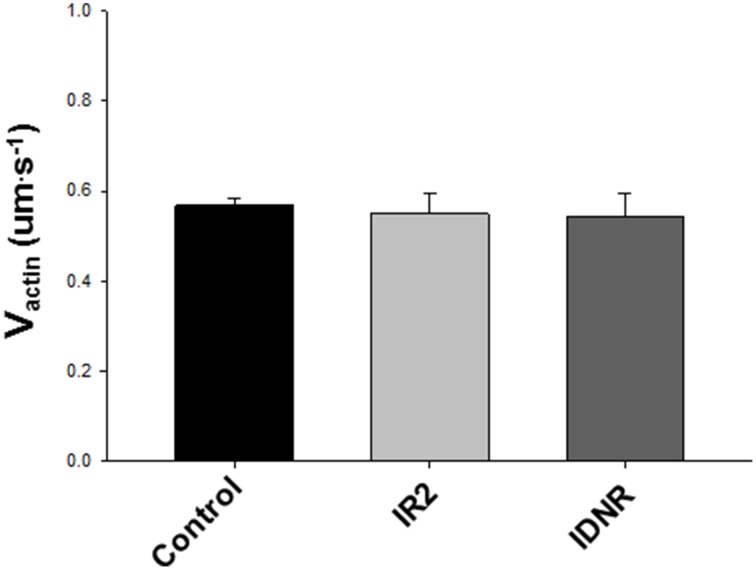
**Effect of ischemia/reperfusion on V_actin_**. Means ± SEM for unregulated (no Tn/Tm) actin filament velocities in the *in vitro* motility assay using myosin isolated from the corresponding condition. The myosin used was isolated from tissue from control, ischemia with 2 h of reperfusion (IR2) and ischemia without resuscitation (IDNR). Data were analyzed using a Kruskal–Wallis ANOVA, but all comparisons were non-significant (*p* > 0.05). The data represent the average actin filament velocities from 106 to 30 s videos from Control, 19 from IR2 and 8, from IDNR.

**Figure 3:**
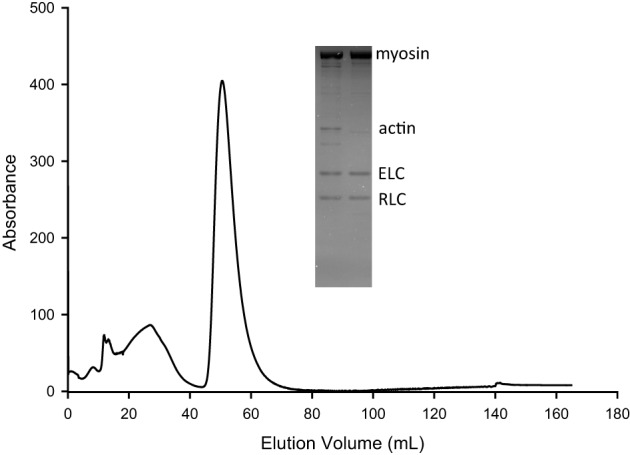
**Representative chromatograph from myosin purification over an HIC column**. The isolated myosin from porcine myocardium was additionally purified using hydrophobic interaction chromotography (HIC) based on a previously described methodology (Malmqvist et al., 2004) with minor modifications (see Methods). Chromatograph displays absorbance at 280 nm vs. elution volume. Proteins with minimal affinity for the column elute at high (1.45 M) AmSO_4_ (first broad peak). After the AmSO_4_ is reduced to 1.2 M myosin is released (large narrow peak). This process removed impurities including actin and likely tropomyosin, selecting for myosin and its light chains (ELC, RLC) as shown by SDS-PAGE gel (inset). In the SDS-PAGE the left lane represents the sample before HIC purification and the right lane after HIC purification.

**Figure 4:**
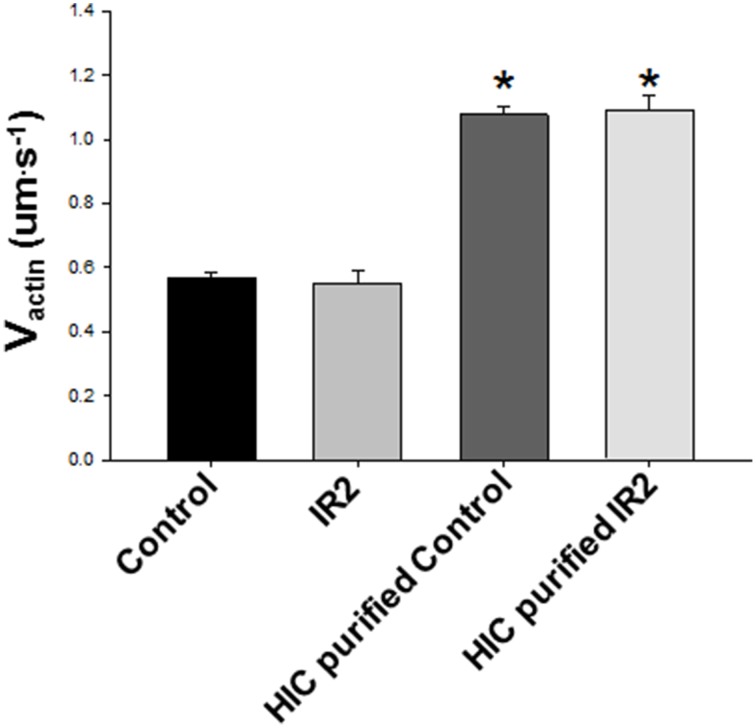
**Purification with HIC column enhances V_actin_**. Further purification of the myosin using an HIC column nearly doubled V_actin_ from both Control tissue and tissue exposed to ischemia (IR2) and 2 h of reperfusion (*p* < 0.001). However, there was no difference between conditions with either the isolated myosin or the HIC purified myosin. The data represent the average actin filament velocities from 106 to 30 s videos from Control and 19 from IR2, as well as five videos each for HIC purified Control and IR2 purified samples. ^*^ Indicates significance at *p* < 0.05.

The ability of cardiac myosin to move unregulated actin filaments in the motility assay was unaffected by the ischemic protocol, whether followed by failed resuscitation (IDNR) or by resuscitation and 2 h of reperfusion (IR2) (Figure 2). Due to the labile nature of cardiac myosin we considered the notion that the ischemic protocol might lead to a reduction in the amount of myosin purified by the HIC column protocol (Figure 3). However, while the myosin purified with an HIC-column moved the actin filaments faster there was no significant difference between myosin for the control vs. either ischemic condition (IR2 and IDNR) (Figure 4). Additionally the amount of released in the early or later absorbance peaks that typically contain myosin that poorly hydrolyze ATP (Malmqvist et al., 2004) were not larger than for the myosin from control tissue. These findings suggest that the present bout of global ischemia did not make the myosin more labile. Overall these findings suggest that the function of myosin was not affected by the ischemic protocol employed in this study. It also suggests that neither the subsequent failed resuscitation IDNR nor the successful resuscitation followed by 2 h of reperfusion IR2 affected actomyosin function.

**Figure 5:**
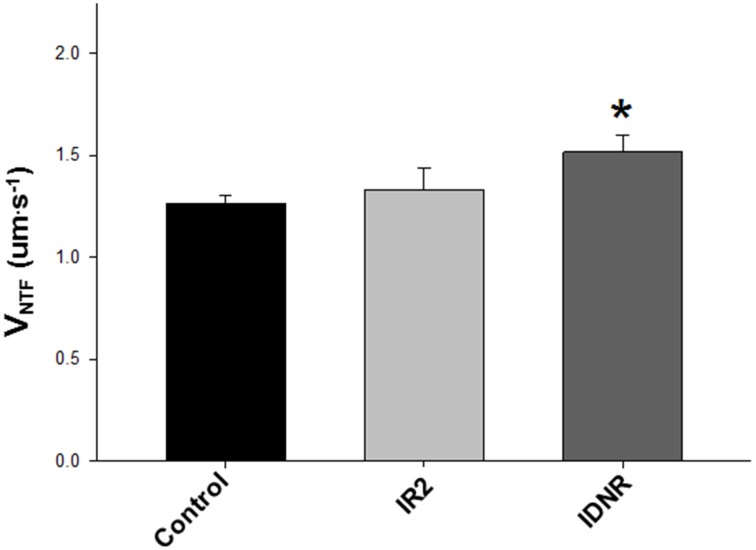
**Effect of ischemia/reperfusion on native thin filament velocities (V_NTF_)**. Means ± SEM for unregulated (no Tn/Tm) actin filament velocities in the *in vitro* motility assay using myosin isolated from the corresponding condition. The myosin used was isolated from control, ischemia/2 h of reperfusion (IR2) and Ischemia without resuscitation (IDNR). Data analyzed using a non-parametric Kruskal–Wallis ANOVA and indicated that IDNR was significantly greater than control velocity ^*^, (*p* < 0.05). The data represent the average actin filament velocities from 20 to 30 s videos from Control, 19 from IR2 and 16 from IDNR.

**Figure 6:**
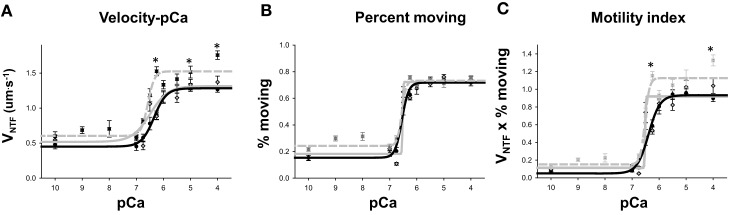
**Velocity-pCa data. (A)** Native thin filament velocity (V_NTF_) plotted as a function of free [Ca^++^] in –log units (pCa). Thin filaments from Control tissue are plotted with filled black dots and solid line, IR2 thin filaments with gray diamonds and solid gray line and the filaments from IDNR are plotted with dark gray boxes and a dashed gray line. Points represent mean ± SEM and the data were fit with the Hill equation (see Methods). ^*^ Indicates significantly (*p* < 0.05) different from WT V_NTF_. **(B)** Percentage of native thin filaments moving as a function of free [Ca^++^] symbols and lines same as in **(A)**. **(C)** Motility index, defined as the product of V_NTF_ and percent moving plotted as a function of free [Ca^++^]. One animal myocardium was used for each condition. The data represent the average actin filament velocities from between 3 and 20 to 30 s videos at each pCa level and for each condition.

The actin binding muscle regulatory proteins troponin and Tm that decorate actin thin filaments are also thought to be vulnerable to modification during a bout of ischemia (Gao et al., 1997; McDonough et al., 1999), therefore we examined the impact of these ischemic perturbations on myosin's ability to translocate native thin filaments (NTF) in an *in vitro* motility assay. Native thin filament velocity (V_NTF_) and calcium sensitivity were unaffected for samples from the IR2 myocardium vs. the control heart (Figures 5, 6 and Table 1). However, native thin filaments from the heart which did not survive resuscitation (IDNR) demonstrated an increase in calcium-sensitivity (pCa_50_ 6.57 ± 0.04 vs. 6.34 ± 0.07 control, Figure 6A and Table 1) and a 25% enhancement (*p* < 0.05) in sliding velocity when fully calcium activated, i.e., pCa 4.0 (Figure 6A). In addition, the Hill coefficient was also increased compared to control filaments, suggesting that myosin binding to these filaments was more cooperative than in the control filaments, but this did not reach statistical significance due to the high variability of this measure under this condition (Table 1). These changes occurred despite a similar percentage of filaments moving in each of the conditions (Figure 6), indicating that the change in average velocity cannot be attributed to a decrease in the number of filaments moving. In contrast, sliding velocities and pCa_50_-values for native thin filament isolated from the heart which was successfully resuscitated and reperfused (IR2) were indistinguishable from the control native thin filaments (Figure 6 and Table 1).

**Table 1:** **Parameters for velocity-pCa fits to the Hill equation**.

**V_NTF_**	**pCa_50_**	***n***
Control	6.34 ± 0.07	1.89 ± 0.48
IR2	6.47 ± 0.13	1.27 ± 0.42
IDNR	6.57 ± 0.04^*^	3.46 ± 1.00

*V_NTF_ indicates native thin filament velocities for filaments isolated from non-ischemic control, ischemia followed by 2 h of reperfusion (IR2) and Ischemia followed by failed resuscitation (IDNR) Values represent mean ± SEM*.

* *Indicates significantly (p < 0.05) different from control tissue. The tissue was obtained from one animal for each condition. The data represent the average actin filament velocities from 20 to 30 s videos from control, 19 from IR2 and 16 from IDNR*.

The significant functional changes to native thin filament velocity over a range of calcium concentrations for the native thin filaments from heart of the animal which did not survive resuscitation (IDNR) suggested underlying structural changes to Tn and/or Tm. We therefore used quantitative mass spectrometry to determine the nature of these changes.

### Quantification of phosphorylation

**Figure 7:**
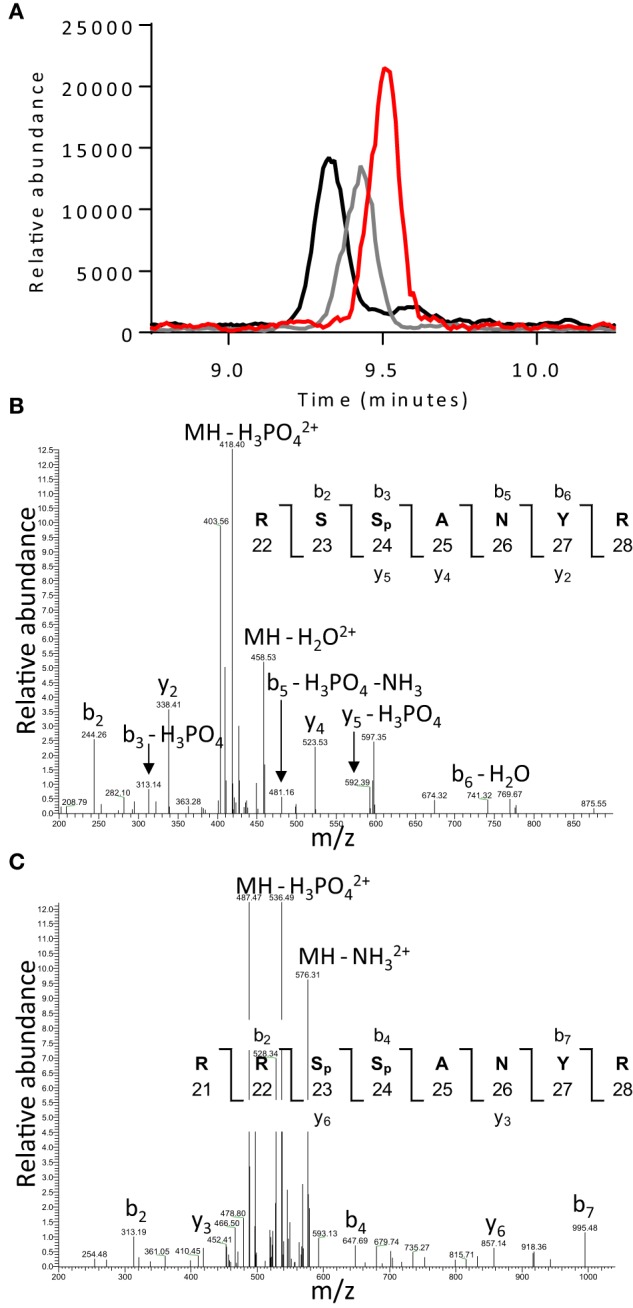
**LC elution profiles and data dependent MS^2^ spectra for troponin I peptides. (A)** Representative LC elution profiles for the non-phosphorylated ^23^SSANYR^28^ (red), mono-phosphorylated ^22^RSSpANYR^28^ (gray), and bis-phosphorylated ^21^RRSpSpANYR^28^ (black) peptides. Data dependent MS^2^ spectra for the **(B)** mono-phosphorylated ^22^RSSpANYR^28^ and **(C)** bis-phosphorylated ^21^RRSpSpANYR^28^ peptides showing fragment ions used for peptide identification and localization of phosphate.

Peptides containing phosphate including: the mono-phosphorylated ^22^RSS_p_ANYR^28^ (m/z = 467.20 R) and bis-phosphorylated ^21^RRS_p_S_p_ANYR^28^ (m/z = 585.23) peptides coming from TnI (Figure 7); and ^269^AISEELDHALNDMTS_p_I^284^ (m/z = 919.89), ^269^AISEELDHALNDm^*^TS_p_I^284^ (m/z = 927.89), and ^269^AISEELDHALNDm∧TS_p_I^284^ (m/z = 935.89) peptides coming from α-Tm were identified in the data dependent MS^2^ spectra and manually confirmed (Previs et al., 2008; Weith et al., 2012). Note, S_p_ represents phosphoserine and m^*^ and m∧denote methionine sulfoxide and sulfone. The degree of phosphorylation at these sites was determined via a mass-balance approach (Previs et al., 2008; Weith et al., 2012) from the abundance of the non-phosphoshorylated analogs of these phosphopeptides in each sample prior to and after the removal of phosphate with alkaline phosphatase. The non-phosphorylated analogs were the properly cleaved ^23^SSANYR^28^ peptide and the sum of the ion currents for the ^269^AISEELDHALNDMTSI^284^, ^269^AISEELDHALNDm^*^TSI^284^, and ^269^AISEELDHALNDm∧TSI^284^ peptides. Whereas this mass-balance approach is an indirect way to measure phosphorylation levels, it provides accurate quantification of the fraction of the protein molecules in a sample which are phosphorylated and obviates problems with quantification arising from mis-cleavage when the phosphate is proximal to the tryptic cleavage site (Previs et al., 2008). For quantification, measured ion currents corresponding to peptides of interest were extracted from the LC chromatogram and the area under each LC peak was normalized using reference peptides within each sample to account for difference in the total amount of protein loaded onto the gel (Previs et al., 2008; Weith et al., 2012). The area under each LC peak for the TnI peptides of interest were normalized using the YDVEAK (m/z = 362.67), KLQLK (m/z = 629.43), ETLDLR (m/z = 746.40), NITEIADLNQK (m/z = 629.83), and IFDLR(m/z = 332.19) peptides; and the area under each LC for α-Tm peptides of interest were normalized using the HIAEDADR (m/z = 463.72), SLEAQAEK (m/z = 438.22), IQLVEEELDR (m/z = 622.33), LVIIESDLER (m/z = 593.83), and SIDDLEDELYAQK (m/z = 769.86) peptides as references.

**Figure 8:**
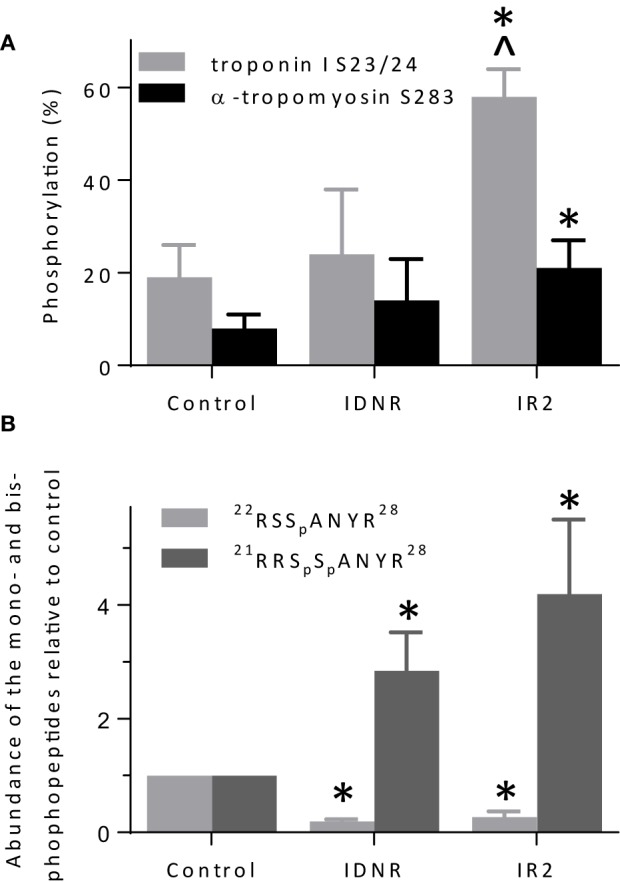
**Quantification of troponin I and α-tropomyosin phosphorylation. (A)** Percent phosphorylation of troponin I serines 23 and/or 24 and tropomyosin serine 283 in native thin filaments isolated from control hearts, and hearts following global ischemia that did not survive attempted resuscitation (IDNR) or survived and were subject to 2 h reperfusion (IR2). **(B)** Levels of mono- (serine 24) and bis-phosphorylated (serines 23 and 24) troponin I relative to the control. Three dephosphorylated control and experimental samples were prepared from each heart. ^*^p < 0.01 relative to control and ^*p* < 0.01 relative to INDR. The difference between IR2 and IDNR was *p* = 0.06 for tropomyosin phosphorylation.

The overall percent phosphorylation of both TnI at serines 23/24 and α-Tm at serine 283 was significantly enhanced with respect to the control (Figure 8A) in samples from successful resuscitation followed by 2 h of reperfusion (IR2). In contrast, the overall levels of TnI or Tm phosphorylation of samples from the failed resuscitation (IDNR) did not significantly differ from the control samples (Figure 8A). However, this method of quantification is not sensitive to changes in site-specific phosphorylation when multiple phosphorylation sites are located within a single peptide, as was the case for TnI serine 23/24. Therefore, we determined if there was a shift in the phosphorylation profile between the mono- and bis-phosphorylated states by calculating the relative abundance of the mono-phosphorylated ^22^RSS_p_ANYR^28^ and bis-phosphorylated ^21^RRS_p_S_p_ANYR^28^ peptides in each sample (Figure 8B). We observed significant reductions in the abundance of the mono-phosphorylated peptides [0.27 ± 0.10 (IR2) and 0.19 ± 0.04 (IDNR)] and corresponding increases in the abundance of the bis-phosphorylated peptides [4.2 ± 1.3 (IR2) and 2.8 ± 0.7 (IDNR)] with respect to the controls following the bout of ischemia regardless of survival (Figure 8B). Therefore, under both ischemic conditions we observed in increase in the relative abundance of the bis-phosphorylated TnI ^21^RRS_p_S_p_ANYR^28^ peptide with respect to the control.

### No truncation of troponin I

One of the most frequently observed structural changes associated with ischemia is the proteolytic cleavage of the C-terminal end of TnI (Westfall and Solaro, 1992; Gao et al., 1997; van Eyk et al., 1998; McDonough et al., 1999; van Eyk and Murphy, 2001). We therefore used a mass spectrometry based, mass-balance approach to determine whether the C-terminus of TnI was truncated during ischemia. We measured the relative abundance of the sum of the C-terminal ^195^NIDALSGMEGR^205^ (m/z = 581.78) and ^195^NIDALSGm^*^EGR^205^ (m/z = 589.77) peptides in the control and experimental samples that would be removed by ischemia induced proteolytic cleavage. The area under the LC peak for the C-terminal peptide was normalized using the TnI reference peptides used for the quantification of phosphorylation. The abundance of the C-terminal peptide did not differ for either group [1.09 ± 0.19 (IR2), 1.06 ± 0.26 (IDNR)] when compared to controls. This lack of difference suggests that the C-terminus of TnI was not truncated as a result of the ischemic bout employed in the present study.

## Discussion

### Effects on the *in vitro* function of the contractile proteins

We examined the structural and functional effects of global cardiac ischemia and reperfusion on the sarcomeric contractile proteins of the myocardium to gain molecular insights into the persistent depression in cardiac function after cardiac arrest and resuscitation. The movement of actin filaments in an *in vitro* motility assays did not differ on cardiac myosin isolated from hearts that were subject to ventricular fibrillation followed by either successful (IR2) or unsuccessful (IDNR) resuscitation (Figure 2). Although we did not attempt to identify structural changes in myosin, these findings indicate that this form of ischemia has no effect on myosin's ability to translocate actin and therefore suggests any modifications do not underlie the persistent depression in contractility following cardiac arrest. This lack of effect of global ischemia and reperfusion on myosin function was somewhat unexpected. Prior studies using more localized models of ischemic, including coronary artery occlusion, showed that both myosin and the thin filament can be modified by the accumulation of reactive oxygen species (Zweier et al., 1989), a putative agent of protein modification and damage from ischemia (Bolli and Marban, 1999). These ROS mediated changes can reduce myosin's ability to hydrolyze ATP (Tiago et al., 2006) and its ability to translocate actin and generate force *in vitro* (Rao et al., 2007), but neither effect was observed in the present study. In comparing the models employed the prior studies utilized more localized and longer duration models of ischemia that lasted for 15 min to 1 h and they reperfused the heart for longer periods of 24–72 h. Therefore, the absence of an effect of ischemia on myosin's function in the present study may be due to the different nature of the ischemia or the shorter duration of either the ischemic bout and/or the subsequent reperfusion. In the present study we used shorter bouts of ischemia (12 min) because longer bouts (>15 min) result in the majority of the animals failing to be resuscitated (Kern et al., 1996), which would strongly limit the ability to study a model that resembles the ROSC after cardiac arrest. The timing employed in the present study also makes the findings more applicable to typical timing experienced during out-of-the-hospital cardiac arrest where the average response time of an emergency medical technician unit is ~8 min from dispatch to arrival (Cobb et al., 1999). Thus, more localized models with longer periods of ischemia and reperfusion may be needed to observe a significant impact on myosin function.

### Modifications to the regulatory proteins

Another unexpected finding based on information for more localized bouts of ischemia was the absence of degradation of TnI. Various models of ischemia result in proteolytic cleavage of the C-terminal end of TnI with the amount cleavage increased by the intensity and severity of the ischemia (Gao et al., 1997; McDonough et al., 1999). The resultant truncation of TnI impacts the function, disrupting the precise interaction of TnI and the calcium binding subunit of troponin, TnC. This interaction is crucial for activation of the filament and its disruption has been implicated as a partial cause of contractile dysfunction following ischemia (Gao et al., 1997; McDonough et al., 1999) by affecting Ca^2+^ sensitivity and force production (Westfall and Solaro, 1992; van Eyk et al., 1998; van Eyk and Murphy, 2001; Foster et al., 2003; Day et al., 2007). Therefore, we expected that TnI would be truncated in the present study, but this was not the case in the present study based on the LC-MS data (Figure 7). Similar to the lack of effect on myosin function described above, the absence of any truncation of TnI may be the result of the shorter duration of ischemia in the present study compared to previous studies. In support of this notion, previous demonstration of this truncation employed bouts of ischemia that ranged from 15 to 60 min, with the most severe truncations occurring with 60 min bouts (Westfall and Solaro, 1992; Gao et al., 1995, 1997; van Eyk et al., 1998). Thus, the type of ischemia elicited by inducing ventricular fibrillation may produce quite different structural and functional changes in the myocardium than other forms of ischemia. This suggests that these two forms of myocardial stunning, while both resulting from a reduction in myocardial blood flow may elicit distinctly different effects on the contractile proteins.

In contrast to the lack of TnI truncation, we observed increases in the level of TnI bis-phosphorylation on serines 23 and 24 in all samples subjected to ischemia regardless of survival (Figure 8B) but we only observed an increase in phosphorylation of α-Tm in the samples from the pigs that survived resuscitation and were reperfused (Figure 8A). Interestingly, thin filament calcium sensitivity and maximal sliding velocities (Figures 5, 6) were only significantly affected by this modification in the native thin filaments isolated from samples where the animal did not survive resuscitation (IDNR). Previous investigations have shown that the effect of ischemia on the phosphorylation level of TnI and α-Tm is equivocal, with some authors observing consistent decreases (Han and Ogut, 2010, 2011) while others have observed no change or increases in phosphorylation (Rao et al., 2007). The discrepancies in previous findings could again result from the different models and durations of ischemia in each study. Our findings suggest that both TnI bis-phosphorylation at serines 23 and 24 and α-Tm phosphorylation at serine 283 may work in parallel to alter calcium sensitivity and maximal sliding velocities. We saw increases in calcium sensitivity and maximal sliding velocities when the level of bis-phosphorylation of TnI was increased in the IDNR samples. However, despite the phosphorylation levels of TnI being even greater in the IR2 samples as compared to the controlsthis gain of function was lost when it occurred in conjunction with an increase in the level of α-Tm phosphorylation. This may arise if TnI phosphorylation alters the ability of Tn to be more sensitive to Ca^++^, while Tm phosphorylation affects end-to-end contacts and cooperative recruitment of myosin molecules to the thin filament (Rao et al., 2009). In the present study the Tm phosphorylation may have offset the increase in Ca^++^-sensitivity mediated by the relative increase in bis-phosphorylation of TnI (Figure 8).

## Conclusions

We induced cardiac arrest in a swine model followed by resuscitation and reperfusion in order to determine if this form of global cardiac ischemia induces changes to the structure and/or function of the contractile proteins of the myocardium. We found that samples from the successful resuscitation and reperfusion (IR2) protocol did not significantly alter the ability of myosin to bind to and move actin, nor the ability of Tn or Tm to regulate or modulate the actomyosin interaction (Figures 5, 6). However, in samples from failed resuscitation (IDNR) we observed a significant increase in the maximum sliding velocity with native thin filaments and an increased sensitivity to Ca^++^ (Figures 5, 6). Since no functional effects were observed using unregulated actin filaments (Figures 2, 4), this suggests that the enhanced V_NTF_ and increased Ca^++^-sensitivity is mediated through the regulatory proteins, Tn, and Tm and not through myosin or actin. To probe the potential mechanism underlying the function changes we used mass spectrometry to identify and quantify modifications to the regulatory proteins. Interestingly, samples from both the IDNR and IR2 hearts showed a significant shift from the mono-phosphorylated to bis-phosphorylated form of TnI while the levels of Tm phosphorylation only significantly increased in the IR2 samples (Figure 8). Previous experiments have shown that treatment of TnI with PKA and reconstitution into thin filaments results in an increase in calcium sensitivity for velocity *in vitro* motility assay (Hunlich et al., 2005) and the results shown here suggest this increase in sensitivity may be negated upon phosphorylation of Tm. Additionally, although TnI phosphorylation may enhance sliding velocity, the previous report showed that TnI phosphorylation decreases calcium sensitivity for force when a load is added to the *in vitro* motility assay. This finding may explain the inconsistencies regarding the role of TnI in altering calcium sensitivity of muscle fiber in disease (Marston and de Tombe, 2008).

While there are likely a multitude of other changes that might contribute to the depressed contractility and poor prognosis following ROSC after cardiac arrest, our findings suggest there are modifications to the phosphorylation status of the thin filament protein that affect the function of the muscle regulatory proteins. It will be important in subsequent studies to understand if and how additional potential changes contribute to the persistent alterations in cardiac contractility in response to ROSC. Limitations of the current study include both a limited number of animals studied and the lack of both structural and functional data concerning the effects ROSC on the thick filament regulatory protein myosin-binding protein C (MyBP-C). Previous studies have shown that changes in MyBP-C content phosphorylation levels can alter calcium sensitivity and maximal sliding velocities (Previs et al., 2012) and that MyBP-C is a target for truncation following cardiac ischemia (Sadayappan, 2012). Thus, this would be a potentially interesting line of inquiry to pursue in future studies.

### Conflict of interest statement

The authors declare that the research was conducted in the absence of any commercial or financial relationships that could be construed as a potential conflict of interest.

## Abbreviations

IDNR: non-successful resuscitation after 20 min
HIC: hydrophobic interaction chromatography
IR2: successful resuscitation followed by 2 h of reperfusion
ROSC: return of spontaneous circulation
Tn: troponin
Tm: tropomyosin
VF: ventricular fibrillation
V_NTF_: native thin filament velocity.

## References

[B1] Allen D. G. Orchard C. H. (1987). Myocardial contractile function during ischemia and hypoxia. Circ. Res. 60, 153–68. 10.1161/01.RES.60.2.153 3552284

[B2] Bolli R. Marban E. (1999). Molecular and cellular mechanisms of myocardial stunning. Physiol. Rev. 79, 609–634. 10221990 10.1152/physrev.1999.79.2.609

[B3] Chalkias A. Xanthos T. (2012). Pathophysiology and pathogenesis of post-resuscitation myocardial stunning. Heart Fail. Rev. 17, 117–128. 10.1007/s10741-011-9255-1 21584712

[B4] Cobb L. A. Fahrenbruch C. E. Walsh T. R. Copass M. K. Olsufka M. Breskin M.. (1999). Influence of cardiopulmonary resuscitation prior to defibrillation in patients with out-of-hospital ventricular fibrillation. JAMA 281, 1182–1188. 10.1001/jama.281.13.1182 10199427

[B5] Day S. M. Westfall M. V. Metzger J. M. (2007). Tuning cardiac performance in ischemic heart disease and failure by modulating myofilament function. J. Mol. Med. 85, 911–921. 10.1007/s00109-007-0181-6 17396243

[B6] Debold E. P. Longyear T. J. Turner M. A. (2012). The effects of phosphate and acidosis on regulated thin filament velocity in an *in vitro* motility assay. J. Appl. Physiol. 113, 1413–1422. 10.1152/japplphysiol.00775.2012 23019317

[B7] Debold E. P. Turner M. A. Stout J. C. Walcott S. (2011). Phosphate enhances myosin-powered actin filament velocity under acidic conditions in a motility assay. Am. J. Physiol. Regul. Integr. Comp. Physiol. 300, R1401–R1408. 10.1152/ajpregu.00772.2010 21346239

[B8] Eisenberg M. S. Mengert T. J. (2001). Cardiac resuscitation. N. Engl. J. Med. 344, 1304–1313. 10.1056/NEJM200104263441707 11320390

[B9] Elliott A. C. Smith G. L. Eisner D. A. Allen D. G. (1992). Metabolic changes during ischaemia and their role in contractile failure in isolated ferret hearts. J. Physiol. 454, 467–490. 10.1113/jphysiol.1992.sp019274 1474498 PMC1175615

[B10] Foster D. B. Noguchi T. van Buren P. Murphy A. M. van Eyk J. E. (2003). C-terminal truncation of cardiac troponin I causes divergent effects on ATPase and force: implications for the pathophysiology of myocardial stunning. Circ. Res. 93, 917–924. 10.1161/01.RES.0000099889.35340.6F 14551240

[B11] Gao W. D. Atar D. Backx P. H. Marban E. (1995). Relationship between intracellular calcium and contractile force in stunned myocardium. Direct evidence for decreased myofilament Ca2+ responsiveness and altered diastolic function in intact ventricular muscle. Circ. Res. 76, 1036–1048. 10.1161/01.RES.76.6.1036 7758158

[B12] Gao W. D. Atar D. Liu Y. Perez N. G. Murphy A. M. Marban E. (1997). Role of troponin I proteolysis in the pathogenesis of stunned myocardium. Circ. Res. 80, 393–399. 9048660

[B13] Han Y. S. Ogut O. (2010). Regulation of fibre contraction in a rat model of myocardial ischemia. PLoS ONE 5:e9528. 10.1371/journal.pone.0009528 20209103 PMC2832002

[B14] Han Y. S. Ogut O. (2011). Force relaxation and thin filament protein phosphorylation during acute myocardial ischemia. Cytoskeleton (Hoboken) 68, 18–31. 10.1002/cm.20491 20925105 PMC3005133

[B15] Hunlich M. Begin K. J. Gorga J. A. Fishbaugher D. E. LeWinter M. M. van Buren P. (2005). Protein kinase A mediated modulation of acto-myosin kinetics. J. Mol. Cell. Cardiol. 38, 119–125. 10.1016/j.yjmcc.2004.10.005 15623428

[B16] Kern K. B. (2002). Postresuscitation myocardial dysfunction. Cardiol. Clin. 20, 89–101. 10.1016/S0733-8651(03)00067-5 11845547

[B17] Kern K. B. Hilwig R. W. Rhee K. H. Berg R. A. (1996). Myocardial dysfunction after resuscitation from cardiac arrest: an example of global myocardial stunning. J. Am. Coll. Cardiol. 28, 232–240. 10.1016/0735-1097(96)00130-1 8752819

[B18] Lehman W. Vibert P. Uman P. Craig R. (1995). Steric-blocking by tropomyosin visualized in relaxed vertebrate muscle thin filaments. J. Mol. Biol. 251, 191–196. 10.1006/jmbi.1995.0425 7643394

[B19] Mader T. J. Kellogg A. R. Walterscheid J. K. Lodding C. C. Sherman L. D. (2010). A randomized comparison of cardiocerebral and cardiopulmonary resuscitation using a swine model of prolonged ventricular fibrillation. Resuscitation 81, 596–602. 10.1016/j.resuscitation.2010.01.013 20176434

[B20] Malmqvist U. P. Aronshtam A. Lowey S. (2004). Cardiac myosin isoforms from different species have unique enzymatic and mechanical properties. Biochemistry 43, 15058–15065. 10.1021/bi0495329 15554713

[B21] Margossian S. S. Lowey S. (1982). Preparation of myosin and its subfragments from rabbit skeletal muscle. Methods Enzymol. 85(Pt B), 55–71. 10.1016/0076-6879(82)85009-X 6214692

[B22] Marston S. B. de Tombe P. P. (2008). Troponin phosphorylation and myofilament Ca2+-sensitivity in heart failure: increased or decreased? J. Mol. Cell. Cardiol. 45, 603–607. 10.1016/j.yjmcc.2008.07.004 18691597 PMC2610448

[B23] McDonough J. L. Arrell D. K. van Eyk J. E. (1999). Troponin I degradation and covalent complex formation accompanies myocardial ischemia/reperfusion injury. Circ. Res. 84, 9–20. 10.1161/01.RES.84.1.9 9915770

[B24] Neumar R. W. Nolan J. P. Adrie C. Aibiki M. Berg R. A. Bottiger B. W.. (2008). Post-cardiac arrest syndrome: epidemiology, pathophysiology, treatment, and prognostication. A consensus statement from the International Liaison Committee on Resuscitation (American Heart Association, Australian and New Zealand Council on Resuscitation, European Resuscitation Council, Heart and Stroke Foundation of Canada, InterAmerican Heart Foundation, Resuscitation Council of Asia, and the Resuscitation Council of Southern Africa); the American Heart Association Emergency Cardiovascular Care Committee; the Council on Cardiovascular Surgery and Anesthesia; the Council on Cardiopulmonary, Perioperative, and Critical Care; the Council on Clinical Cardiology; and the Stroke Council. Circulation 118, 2452–2483. 10.1161/CIRCULATIONAHA.108.190652 18948368

[B25] Previs M. J. Beck P. S. Gulick J. Robbins J. Warshaw D. M. (2012). Molecular mechanics of cardiac myosin-binding protein C in native thick filaments. Science 337, 1215–1218. 10.1126/science.1223602 22923435 PMC3561468

[B26] Previs M. J. van Buren P. Begin K. J. Vigoreaux J. O. LeWinter M. M. Matthews D. E. (2008). Quantification of protein phosphorylation by liquid chromatography-mass spectrometry. Anal. Chem. 80, 5864–5872. 10.1021/ac800337v 18605695 PMC3050605

[B27] Rao V. S. La Bonte L. R. Xu Y. Yang Z. French B. A. Guilford W. H. (2007). Alterations to myofibrillar protein function in nonischemic regions of the heart early after myocardial infarction. Am. J. Physiol. Heart Circ. Physiol. 293, H654–H659. 10.1152/ajpheart.01314.2006 17400716

[B28] Rao V. S. Marongelli E. N. Guilford W. H. (2009). Phosphorylation of tropomyosin extends cooperative binding of myosin beyond a single regulatory unit. Cell Motil. Cytoskeleton 66, 10–23. 10.1002/cm.20321 18985725 PMC2770177

[B29] Roger V. L. Go A. S. Lloyd-Jones D. M. Benjamin E. J. Berry J. D. Borden W. B.. (2012). Heart disease and stroke statistics–2012 update: a report from the American Heart Association. Circulation 125, e2–e220. 10.1161/CIR.0b013e31823ac046 22179539 PMC4440543

[B30] Sadayappan S. (2012). Cardiac myosin binding protein-C: a potential early-stage, cardiac-specific biomarker of ischemia-reperfusion injury. Biomark. Med. 6, 69–72. 10.2217/bmm.11.100 22296197 PMC3339268

[B31] Schaefer S. Schwartz G. G. Gober J. R. Wong A. K. Camacho S. A. Massie B.. (1990). Relationship between myocardial metabolites and contractile abnormalities during graded regional ischemia. Phosphorus-31 nuclear magnetic resonance studies of porcine myocardium *in vivo*. J. Clin. Invest. 85, 706–713. 10.1172/JCI114495 2312722 PMC296486

[B32] Schoenenberger R. A. von Planta M. von Planta I. (1994). Survival after failed out-of-hospital resuscitation. Are further therapeutic efforts in the emergency department futile? Arch. Intern. Med. 154, 2433–2437. 10.1001/archinte.1994.00420210069008 7979839

[B33] Tiago T. Simao S. Aureliano M. Martin-Romero F. J. Gutierrez-Merino C. (2006). Inhibition of skeletal muscle S1-myosin ATPase by peroxynitrite. Biochemistry 45, 3794–3804. 10.1021/bi0518500 16533063

[B34] Tyska M. J. Hayes E. Giewat M. Seidman C. E. Seidman J. G. Warshaw D. M. (2000). Single-molecule mechanics of R403Q cardiac myosin isolated from the mouse model of familial hypertrophic cardiomyopathy. Circ. Res. 86, 737–744. 10.1161/01.RES.86.7.737 10764406

[B35] van Eyk J. E. Murphy A. M. (2001). The role of troponin abnormalities as a cause for stunned myocardium. Coron. Artery Dis. 12, 343–347. 10.1097/00019501-200108000-00002 11491198

[B36] van Eyk J. E. Powers F. Law W. Larue C. Hodges R. S. Solaro R. J. (1998). Breakdown and release of myofilament proteins during ischemia and ischemia/reperfusion in rat hearts: identification of degradation products and effects on the pCa-force relation. Circ. Res. 82, 261–271. 10.1161/01.RES.82.2.261 9468197

[B37] Weith A. E. Previs M. J. Hoeprich G. J. Previs S. B. Gulick J. Robbins J.. (2012). The extent of cardiac myosin binding protein-C phosphorylation modulates actomyosin function in a graded manner. J. Muscle Res. Cell Motil. 33, 449–459. 10.1007/s10974-012-9312-y 22752314 PMC3522762

[B38] Westfall M. V. Solaro R. J. (1992). Alterations in myofibrillar function and protein profiles after complete global ischemia in rat hearts. Circ. Res. 70, 302–313. 10.1161/01.RES.70.2.302 1531186

[B39] Zweier J. L. Kuppusamy P. Williams R. Rayburn B. K. Smith D. Weisfeldt M. L.. (1989). Measurement and characterization of postischemic free radical generation in the isolated perfused heart. J. Biol. Chem. 264, 18890–18895. 2553726

